# Cyclic Fatigue Resistance and Force Generated by OneShape Instruments during Curved Canal Preparation

**DOI:** 10.1371/journal.pone.0160815

**Published:** 2016-08-11

**Authors:** Zhuyu Wang, Wen Zhang, Xiaolei Zhang

**Affiliations:** 1 Department of Operative Dentistry and Endodontics, Guanghua School of Stomatology, Sun Yat-sen University, Guangzhou, China; 2 Department of Periodontology and Endodontology, Graduate School of Dentistry, Tohoku University, Sendai, Japan; 3 Guangdong Provincial Key Laboratory of Stomatology, Guangzhou, China; University of Brescia, ITALY

## Abstract

**Objectives:**

To evaluate the cyclic fatigue resistance and the force generated by OneShape files during preparation of simulated curved canals.

**Methods:**

Six OneShape files (the test) and six ProTaper F2 files (the control) were subject to the bending ability test. Another thirty files of each type were used to prepare artificial canals (n = 60), which were divided into 3 groups according to respective curvatures of the canals (30°, 60°, and 90°). The numbers of cycles to fatigue (NCF) as well as the positive and negative forces that were generated by files during canal preparation were recorded. The scanning electron microscopy was applied to detect the fracture surfaces.

**Results:**

Compared with ProTaper F2 files, the bending loads of OneShape files were significantly lower at deflections of 45°(*P* < .05), 60° (*P* < .05) and 75° (*P* < .01). No significant difference was found at 30°. OneShape files presented a higher NCF in both 60° and 90° canals than the control (*P* < .01). No significant difference of NCF was found between OneShape and ProTaper files in 30° canals. During the preparation of 30° canals by both files, the negative forces were dominant. With the increase of the curvature, more positive forces were observed. When the OneShape Files were compared with the control, significant different forces were found at D3 and D2 (*P* < .05) in 30° canals, at D2 (*P* < .05), D1 (*P* < .01) and D0 (*P* < .01) in 60° canals, and at D4 and D3 (*P* < .01) in 90° canals.

**Conclusions:**

OneShape files possessed a reliable flexibility and cyclic fatigue resistance. According to the assessments of the forces generated by files, OneShape instruments performed in a more fatigue-resistant way during curved canal preparation, compared with the ProTaper F2 files.

## Introduction

Nickel-titanium (NiTi) instruments, essentially owning to their superelastic characters, have become an elementary implement in root canal preparation [1, 2]. In spite of the increased flexibility compared to the stainless steel instruments, NiTi instruments are still exposed to the risk of fracture in clinical situations, which was caused by torsional and cyclic fatigues [3, 4]. The cyclic fatigue typically happens in preparing curved root canals where the instruments are subjected to alternating tensile and compressive stresses [5–7]. Nearly 70% NiTi rotary instrument fractures were caused by cyclic fatigue [8]. The evaluation of cyclic fatigue resistance includes the number of cycles to fatigue (NCF) that an instrument is able to endure when rotating in curved canals, and the bending ability which refers to the instrument flexibility [1, 2].

In most cases, the longitudinal axial stress subject to files during canal preparation was considered as resistance resulting from the apical compressive force, which was also named as the positive force (PF) [9, 10]. Contrary to the positive force, the negative force (NF) was caused by the so-called screw-in/threading-in effect [10, 11]. Nevertheless, the characterization of both PF and NF generated during preparation of curved canals is not fully understood.

The single-file system, a new concept for canal preparation, has been introduced recently. OneShape (Micro Méga, Besançon, France), a typical single-file instrument, is fabricated with the conventional austenite 55-NiTi alloy, motor-driven in continuous rotation. Bürklein [12, 13] and Dagna [14] reported that OneShape files possessed an excellent shaping ability and cleaning efficiency. However, the knowledge gap still exists concerning the cyclic fatigue resistance and the force generated by OneShape files in preparing curved canals [15].

The purpose of this *in vitro* study was to evaluate the cyclic fatigue resistance of OneShape instruments, and to assess the positive/negative force generated during preparation of curved canals.

## Materials and Methods

**Fig 1:**
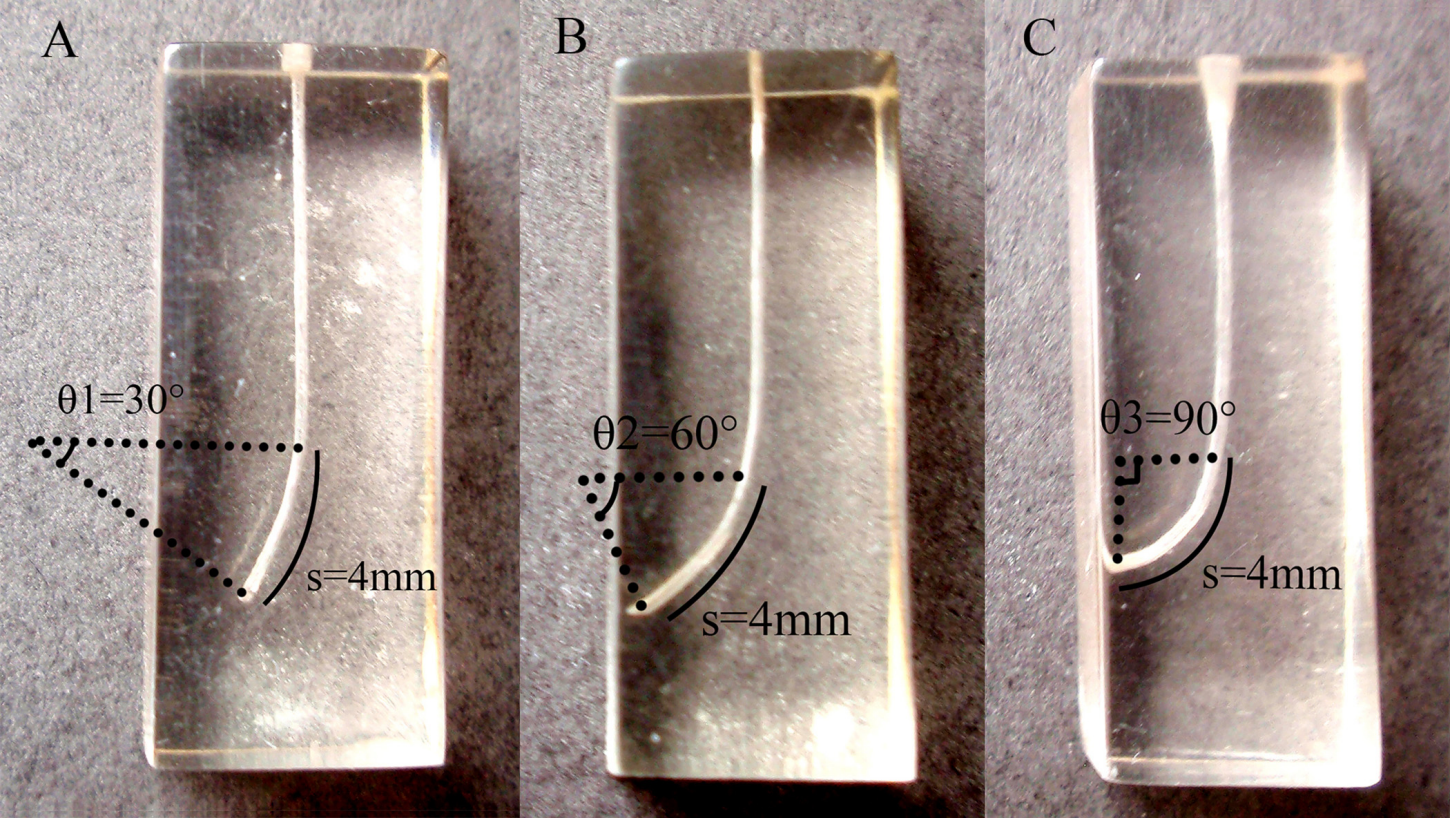
The simulated curved canals. The length of the simulated canal is 16mm. The arc length was 4mm and the curvature was made into 30° (A), 60° (B) and 90° (C), according to the Arc Length Formula (*s* = (*π*×*r*×*θ*)/180).

Thirty-six OneShape single-file instruments (Micro Méga, Besançon, France) and thirty-six ProTaper F2 instruments (Dentsply Maillefer, Ballaigues, Switzerland) (the control) were used in this study. The simulated curved canals were custom-made in plastic blocks (Knoop hardness = 62, KHN) with the inner diameter of 0.2 mm and the total length of 16 mm (HST, Hebei, China). The simulated canals (n = 60) were custom-made into 3 curvatures, *i*.*e*. 30°, 60° and 90°. The configuration of the curved canal was based on the Arc Length Formula, *i*.*e*. *s* = (*π*×*r*×*θ*)/180. *S* is the arc length, *π* is pi (≈3.14), *r* is the radius of the circle, θ is the central angle of the arc in degrees. The arc length (*s*) is set at 4 mm in the apical portion of the canal, the 3 canal curvatures (θ) was then defined and made into 30°, 60° and 90° (Fig 1).

For the OneShape group (the test), the file size was #25 with a constant 0.06 taper (0.25 mm tip diameter). For the ProTaper group (the control), the selected file was F2 file, with a 0.08 taper at its apical 3 mm, followed by a 0.06 taper from the 3 mm up to the coronal. The tip diameter of ProfTaper F2 file is 0.25 mm.

### Bending Ability Test

The bending resistance test, according to the ISO 3630–1 (International Organization for Standardization, 1992), was applied to 6 new instruments of each group [2, 16]. The files were fixed at 3 mm from the tips and were bended from 0° to 75° along the longitudinal axis, using the Universal Test Machine (Electroplus E3000, Instron, USA). The relationship of load to bend was drawn and the force values were recorded at 4 points corresponding to the positions of 30°, 45°, 60° and 75°.

### Cyclic Fatigue Resistance Test

Thirty OneShape files and thirty ProTaper F2 files were used to prepare sixty simulated curved canals, which were divided into three groups according to their respective arc curvature (*i*.*e*. 30°, 60° and 90°). Canals were initially explored with 15# K-file, lubricated with EDTA and flared by the ProTaper SX file (Dentsply Maillefer, Ballaigues, Switzerland). As recommended by the manufacturers, a 16:1 reduction handpiece powered by a torque-controlled motor (X-Smart, Dentsply Maillefer, Switzerland) was applied. The torque and rotational speed was set at 4.0 Ncm, 400 rpm for OneShape, and 2.0 Ncm, 250 rpm for ProTaper F2. The speed of insertion was set at 3 mm/min. After the working length (16 mm) was reached, the files were kept rotating freely in the canals. The time was recorded when the fracture was detected. The NCF was calculated according to the time and rotation rate.

### Positive/Negative Force Detection

The detection of positive/negative force were performed with the Universal Test Machine, as described previously [6]. In brief, during the cyclic fatigue resistance tests when the files were preparing the simulated canals in, both the handpiece and simulated canals were fixed by a rigid holder which was connected to a strain gauge attached to a sensor. The forces generated during the files insertion and canal preparation were simultaneously registered by the sensor. The forces were specifically recorded at 6 locations, *i*.*e*. when the files arrived at 5 mm from working length (D5), 4 mm from working length (D4), 3 mm from working length (D3), 2 mm from working length (D2), 1 mm from working length (D1) and the point of the full working length (D0).

### Scanning Electron Microscopic Observation

After the cyclic fatigue test, the fracture surfaces of fragments were observed under the scanning electron microscope (Quanta 200, FEI, Netherland) for the topographic features analysis.

### Statistics

Data were reported as the mean±standard deviation. The data were analyzed by the analysis of variance and *t*-test (SPSS 19.0, IBM SPSS Statistics, Armonk, USA) with the significance set at *P* < 0.05.

## Results

**Fig 2:**
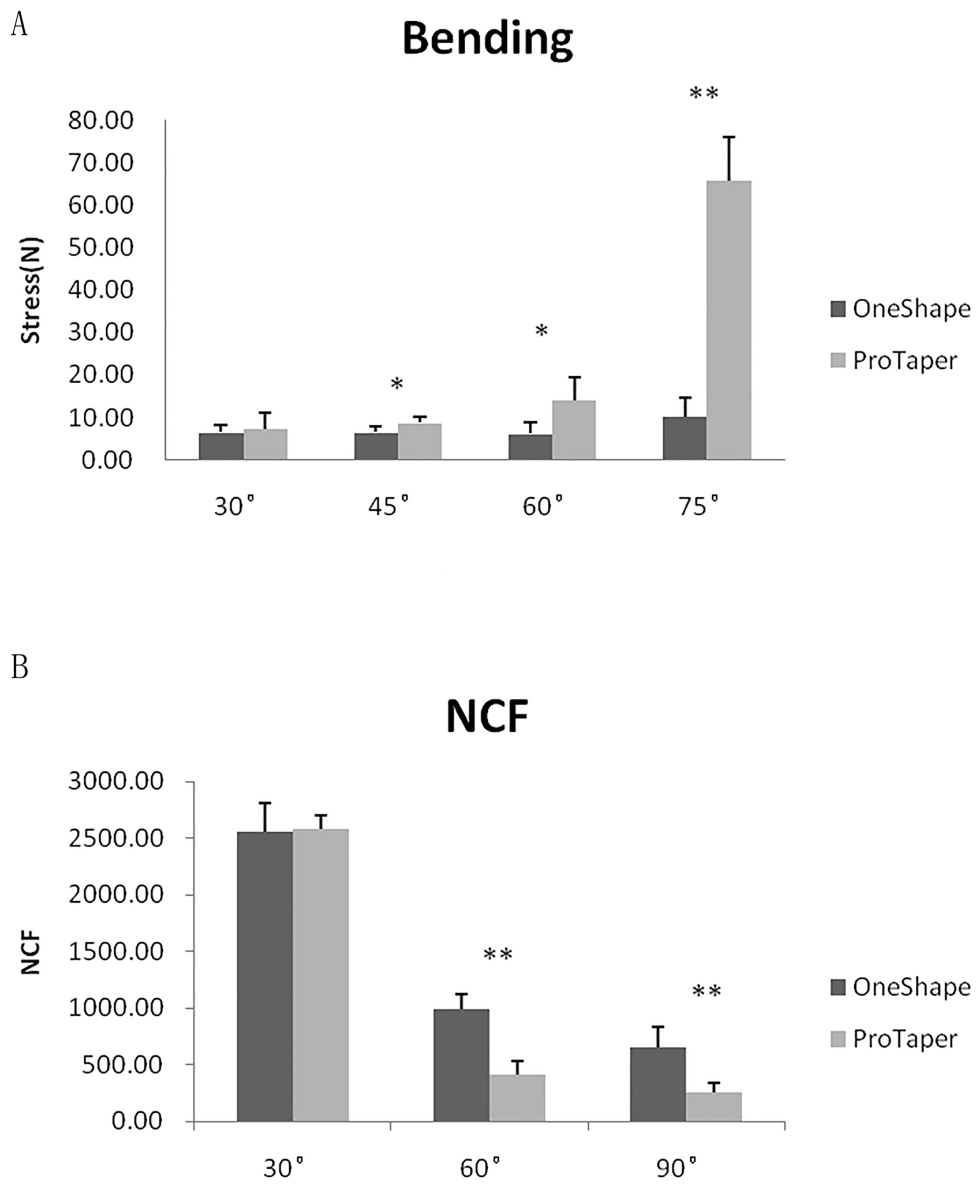
The flexibility evaluation of OneShape files. Compared with ProTaper files (the control), OneShape files presented significantly lower bending values at deflections of 45° (*P* < .05), 60° (*P* < .05) and 75° (*P* < .01) (A). OneShape files presented a higher NCF in both 60° and 90° canals than the control (P < .01). No significant difference of NCF was found between OneShape and ProTaper files in 30° canals. (B).

Compared with the control, the bending load values for OneShape were significantly lower at deflections of 45° (8.83±1.17 *vs*. 6.58±1.28 N), 60° (14.00±5.59 *vs*. 6.17±2.64 N) (*P* < .05) and 75° (65.83±10.21 *vs*. 10.25±4.33 N) (*P* < .01). No significant difference was found at deflections of 30° (7.33±3.93 *vs*. 6.50±1.64 N) (Fig 2A).

The cyclic fatigue resistance test revealed that, when compared with ProTaper F2 files, OneShape files presented a significantly higher NCF in groups of 60° (257.04±82.28 *vs*. 986.18±138.16) and 90° (257.04±82.28 *vs*. 649.88±183.76) (*P* < .01). No significant difference was found in group of 30° (2581.00±121.79 *vs*. 2554.80±254.17) (Fig 2B).

**Fig 3:**
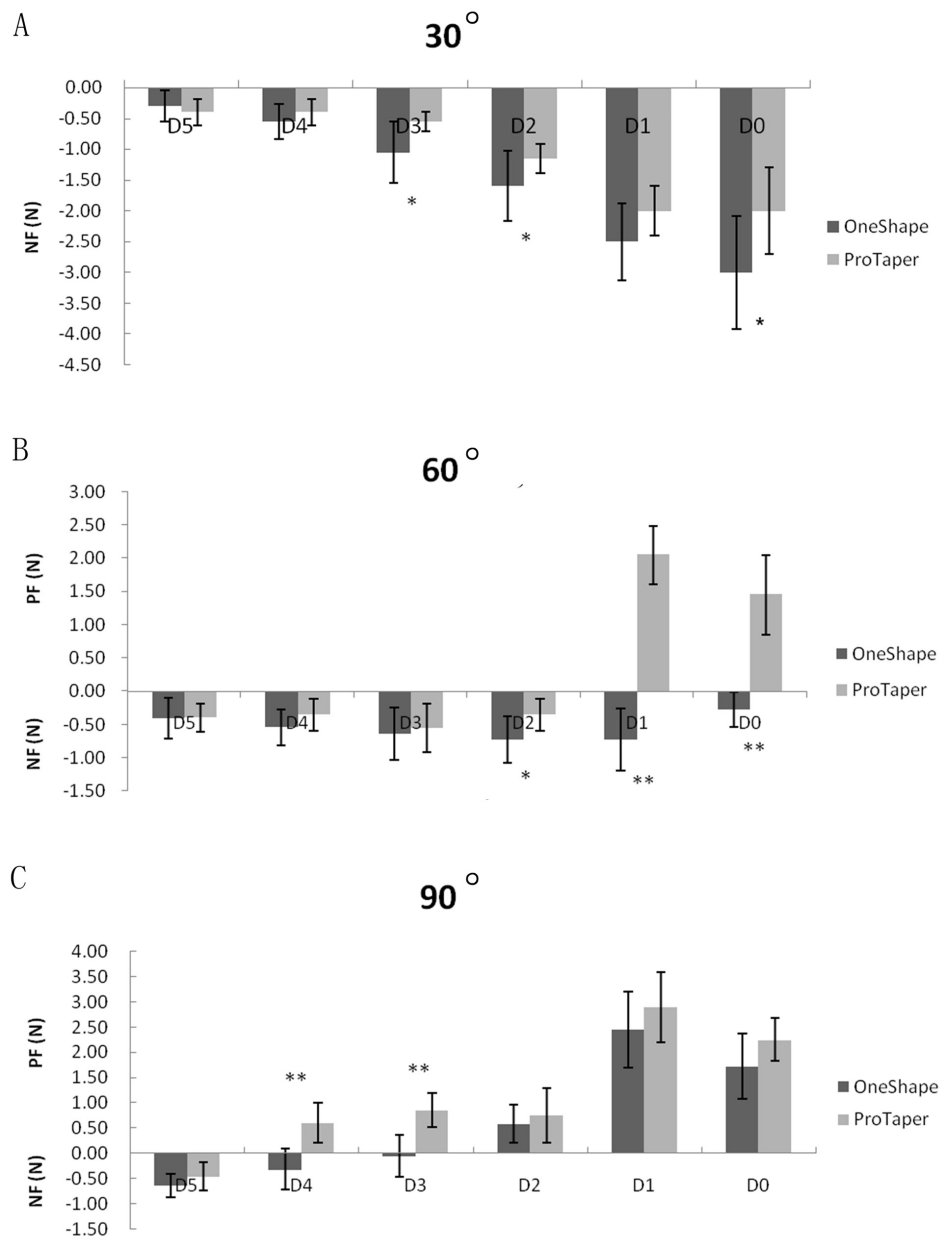
The force generated by OneShape and ProTaper instruments during simulated canal preparation. In group of 30° canals, the negative force generated by OneShape was significantly higher at D3 and D2 (*P* < .05) (A). In group of 60°, the negative force generated by OneShape was significantly higher at D2 (*P* < .05), D1 and D0 (*P* < .01), while the positive force generated by ProTaper F2 was significantly higher at D1 and D0 (*P* < .01) (B). In group of 90°, the negative force generated by OneShape was significantly higher at D4 and D3 (*P* < .01), while the positive force generated by ProTaper F2 was significantly higher at D4 and D3 (*P* < .01) (C). (*: *P* < .05, **: *P* < .01)

As showed in Fig 3, the force generated by OneShape and ProTaper F2 was recorded during simulated canal preparation at the curve of 30°, 60°, and 90° respectively. In group of 30° (Fig 3A), the negative force was dominated for both file types. The value of NF increased with the depth of the prepared canals (from D5 to D0). Compared with the control, the NF generated by OneShape files was significantly higher at D3 (-0.55±0.16 *vs*. -1.05±0.50 N) (*P* < .05) and D2 (-1.15±0.24 *vs*. -1.60±0.57 N) (*P* < .05). In group of 60° (Fig 3B), the NF generated by OneShape files was dominated throughout the recorded canal points. The force generated by ProTaper F2 was negative from D5 to D2 and positive at D1 and D0. When the forces generated by ProTaper F2 *vs*. OneShape files were compared, the significant difference was found at D2 (-0.35±0.24 *vs*. -0.75±0.34 N) (*P* < .05), D1 (2.05±0.44 *vs*. -0.75±0.47 N) (*P* < .01) and D0 (1.45±0.60 *vs*. -0.30±0.26 N) (*P* < .01), respectively. In group of 90° (Fig 3C), the NF generated by OneShape could be observed from D5 to D3 in a decreasing tendency, while the NF generated by ProTaper F2 was only found at D5. In comparison of the forces generated by ProTaper F2 *vs*. OneShape files, the significant difference was found at D4 (0.60±0.39 *vs*. -0.30±0.40 N) (*P* < .01) and D3 (0.85±0.34 *vs*. -0.05±0.42 N) (*P* < .01).

**Fig 4:**
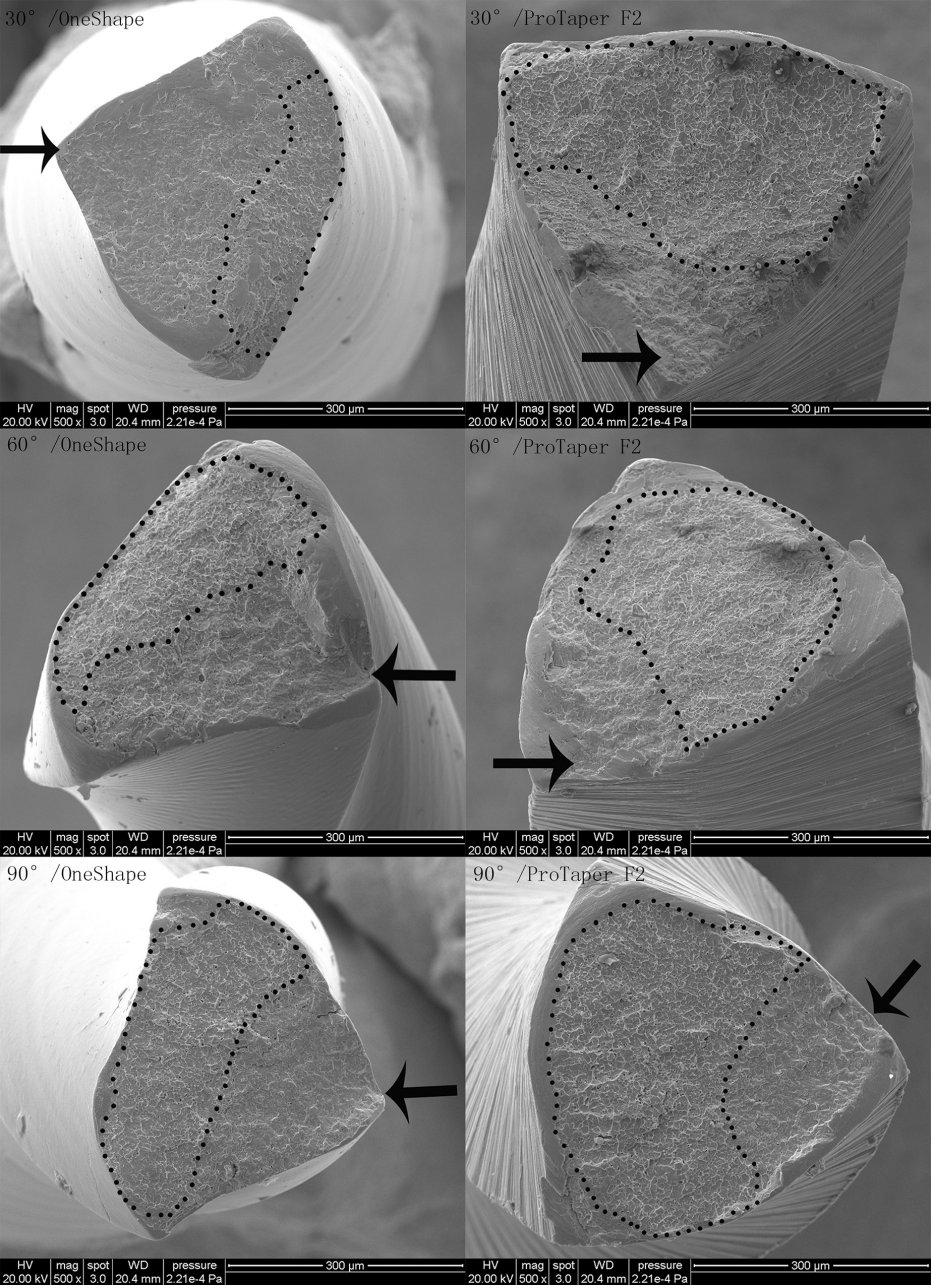
SEM images of the fracture surfaces. The crack initiation areas were pointed by the arrows and the fast fracture zones were surrounded by black dots.

The scanning electron microscopic topography of the fracture surfaces showed typical features of cyclic fatigue. Representative fracture surface images of both OneShape and ProTaper F2 files from the curved canals were lay out in Fig 4. The crack initiation areas were pointed by arrows and the fast fracture zones were highlighted by dotted lines.

## Discussion

Simulated root canals made of plastic blocks were used in the present study, which is similar to previous experiments [17, 18]. It has been reported that during the preparation of the simulated curved canals, the file was subject to the stress similar to the preparation of mandibular incisors [19]. Based on the fact that the Knoop hardness of the material (*i*.*e*. 64 KHN) is close to that of dentin (*i*.*e*. 68 KHN), the plastic blocks were chosen to make simulated canals for this study [20]. The reason to choose simulated canals, instead of canals of extracted teeth, for this *in vitro* experiment is to make a comparable and stable working field to assess the performance of the objective files. ProTaper F2 files were selected as the control because (1) both OneShape and ProTaper F2 files are made of a conventional NiTi alloy, (2) they have the same tip size, (3) they have the similar triangular cross-section design in the tip area and (4) they are applied in continuous clockwise rotation.

The bending ability test was to evaluate the flexibility of the files, which plays a significant role in resisting cyclic fatigue. The flexibility of ProTaper F2 has been studied elsewhere. The results of the present study were similar to those reported by Viana [16] and Necchi [21]. However, no relevant studies on OneShape files were available. The less bending load for OneShape file at deflections of 45° suggested that OneShape file was more flexible than ProTaper F2. The higher flexibility of OneShape file may due to its lower modulus of elasticity and superelastic character, which is also associated with the stress-induced martensitic transformation from austenitic structure [16]. Although both files were made from conventional austenite alloy, OneShape files required lower stress to induce martensitic transformation, indicating the martensite transformation start temperature of OneShape was higher than that of ProTaper F2 [22].

The cyclic fatigue resistance of ProTaper F2 has been reported previously [23, 24], while few study had evaluated the NCF of OneShape file. The results of NCF of OneShape files in the present study were higher than that reported by Capar *et al*. [15], probably due to the different mechanical property of the simulated canals (*i*.*e*. the plastic block of this study *vs*. stainless steel block). Nevertheless, the results of NCF suggested that OneShape files possess better cyclic fatigue resistance than ProTaper F2. A Unique technique of electropolishing procedure applied to the manufacture of OneShape files offered an explanation [25]. This procedure can improve the smoothness of the file surface, and therefore can enhance the cutting efficiency and fatigue resistance [26, 27]. Moreover, OneShape and ProTaper F2 files possess different cross-section diameter because of their different taper. Compared with the OneShape file, the larger cross-section size of the ProTaper F2 file can result in higher stiffness. This will inherently influence the results of the cyclic fatigue resistance test. However, according to Nakagawa *et al* [28], the relationship between cross-sectional area and cyclic fatigue resistance was variable for different NiTi file systems. It’s reasonable to believe that the cross-section or taper of the file alone could not determine the flexibility of the file.

In the present study, the files are driven into the canals in a ramp-like pattern to obtain the smooth and complete depth-stress curve, and longitudinal axial stress was assessed separately as positive force and negative force. Acting as the resistance of the canal wall against files, the increased positive force can lead to longitudinal defects of instruments, accelerating the occurrence cyclic fatigue. On the other hand, the sudden occurrence of screw-in/threading-in effect, which was not observed in present study, could result in increased torque [29]. However, a moderate negative force did benefit the preparation since it was able to drag the files into the canals gradually, decreasing the resistance of preparation (acting as negative resistance) and reducing the possibility of longitudinal defects [10]. During the preparation of the curved canals, the general data suggested that ProTaper F2 received higher positive force and OneShape files were subject to higher negative force. Again in consistent with the findings of NCF, these results revealed that OneShape files possessed better cyclic fatigue resistance. In addition, as suggested by a previous study, a higher negative force reflected better cutting efficiency of a file [25]. The higher negative force of the OneShape file was probably owing to its special cross-section design, including a triangle-shaped symmetrical three-cutting-edge in the tip area, a S-shaped symmetrical two-cutting-edge at the coronal area, and a asymmetrical progressively changing in the middle [30]. The design was alleged to eliminate binding of the instrument in continuous rotation [31].

Due to the resistance of the simulated canals increased along with the curvatures, the negative force decreased along with the increase of the canal curvature [32]. For ProTaper F2, the positive force started to take over in group of 60°. While for the OneShape file, the negative force still dominated until 90° canals. Considering the protective effect of negative force as resistance against cyclic fatigue, it contributed to the higher NCF of OneShape files in curved canals as well.

Scanning electron microscopic observations indicated that both the OneShape and ProTaper F2 showed the typical fracture surfaces of ductile type, consisting of the crack initiation area, fatigue striations, and a fast fracture zone with several dimples and micropores.

## Conclusions

OneShape files possessed a reliable flexibility and cyclic fatigue resistance. According to the assessments of the forces generated by files, OneShape instruments performed in a more fatigue-resistant way during curved canal preparation, compared with the ProTaper F2 files.
